# Condyloma acuminata in child end laser therapy: a case report

**DOI:** 10.1186/1757-1626-2-123

**Published:** 2009-02-04

**Authors:** Mybera Ferizi, Antigona Gercari, Laura Pajaziti, Ymrane Blyta, Allma Kocinaj, Shkendije Dobruna

**Affiliations:** 1 University Clinical Center of Kosovo, Department of Dermatology and Venerology, Prishtine, Republic of Kosovo

## Abstract

**Background:**

Condyloma acuminata are soft, skin colored, fleshy warts that are caused by the Human Papilloma Virus (HPV). The disease is highly contagious, can appear singly or in groups, small or large. The incubation period may be from 1–6 months. Although anogenital warts are considered to be sexually transmitted in adults, this may not be the case for children. Genital warts in children may result from several modes of transmission: from the maternal genital tract autoinoculation, from finger warts and nonsexual transmission from members/careers.

**Case presentation:**

The presented case is a 13-month-old girl. She was hospitalized at the Clinic of Dermatovenerology in 2001 due to papillomatosis changes on the genital area. The changes had started to appear in the sixth month of life, light purple in color, smooth and combined in a tumorous mass, in the vulva and anal areas.

**Conclusion:**

From this case we can come to the conclusion that condyloma acuminate are not only transmitted sexually but through nonsexual ways as well, such is this case, from the infected mother to the infant. Laser therapy is preferred when multiple warts are spread over a large area as though in our case.

## Background

Venereal warts are caused by the human papilloma virus (HPV) which is a small DNA virus that does not grow in tissue culture but can be identified by electron microscopy [1].

The incubation period may take from one to six months [2].

Although anogenital warts are considered to be sexually transmitted in adults, this may not be the case for children [3]. Genital warts at children may result from several modes of transmission: from perinatal transmission of HPV, occurring in uteri and during passage of the neonate through an HPV-infected birth canal, from finger warts and nonsexual transmission from members/careers.

However, the possibility of sexual abuse must always be borne by mind [4].

The topical treatment of childhood condylomata acuminate includes the application of caustic or irritating agents, such as liquid nitrogen trichloroacetic acid or podophyllum resin. These agents poorly tolerated by a child who may require multiple applications. Vaporization of the warts with a carbon dioxide laser is the newest treatment method. The advantage of using the CO2 laser is the ability to treat a large area without causing a scar, stricture, or narrowing of the lumen. This method tends to eliminate the virus and promote rapid healing with very little scar formation[5].

## Case presentation

**Figure 1:**
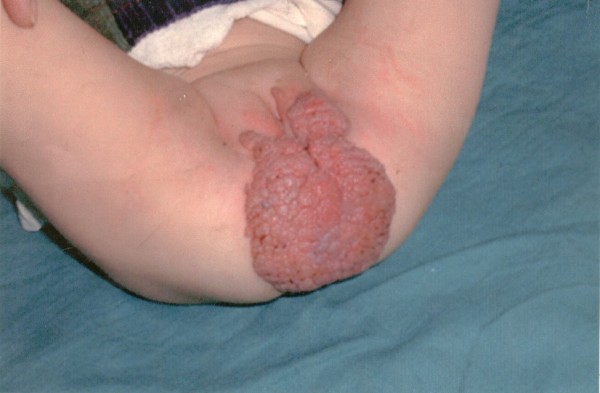
**Multiple warty as a tumor mass of the female genitalia**.

We present a 13-month-old female patient. She was brought to the outpatient department in Dermatological Clinical Center by her mother with a 7 month history of papillomatosis changes in the anogenital area. The changes had started to appear in the sixth month of life, light purple in color, smooth and combined in a tumor mass, in the vulva and anal areas (Figure 1).

The child had been born by vaginal delivery after full term normal pregnancy. From the mother's hetero-anamnesis we learned that the mother has had genital warts during her pregnancy, which have faded out right after the delivery, without previous therapy.

Serological tests for syphilis in the infant and her mother gave negative results.

Based on the heteroanamnesis from her mother, clinical presentation, laboratory and serological tests for syphilis we made a final diagnosis of condylomata acuminate. After general anesthesia our patient is treated with a carbon dioxide laser. This method is well tolerated and promotes rapid healing with very little scar formation.

## Discussion

Sexually transmitted diseases affect the sexually active population being in the reproductive age group and usually are being transmitted in venereal form [2]. Many researches report on big percentage of the infections with HPV at pregnant women [6].

Pregnant women infected with genital warts can pass them on to their newborns [7].

Generally, in children less than 2 years of age, the mode of transmission is vertical from mother to child during childbirth[8]. There is an increasing prevalence of the genital warts in the first three months of the gravidity, which considerably decreases after delivery [8]. Beside the mental-health problems caused by traumatic experiences in the childhood, the sexual abuse can have later influence on the children's growth and development that demands an early and continuous care [7]. It is postulated that the incubation period may be markedly prolonged in cases where sexual abuse is not suspected or found [9]. The increasing incidence of genital warts in adults that has been observed in recent years probably provides a widespread source of infection and results in an increase in the incidence of similar lesions in children. Treatments of genital warts in children have included topical sulfisoxazole, 5% ammoniated mercury ointment and fulguration, cryotherapy and immunotherapy with auto vaccines.

Laser therapy is preferred when multiple warts are spread over a large area, also useful for treating cervical and vaginal warts, when surgical excision is not possible or would be difficult [10]. As malignant transformation of genital warts is known to occur, it is of the utmost importance that such lesions in children be treated promptly[11,12].

## Competing interests

The authors declare that they have no competing interests.

## Authors' contributions

MF admit the patient in the clinic, definite the diagnosis and consult the children and plastic surgeon for treatment. AG and LP was a major contributor in writing the manuscript. All together decide for laser treatment. All authors read and approved the final manuscript.

## Consent

Written informed consent was obtained from the patient for publication of this case report and accompanying images. A copy of the written consent is available for review by the Editor-in-Chief of this journal.
